# Cruelty toward Dogs and Cats in the Republic of Serbia during a 10-Year Period

**DOI:** 10.3390/ani14131926

**Published:** 2024-06-29

**Authors:** Jelena Aleksic Radojkovic, Vladimir Nesic, Anja Ilic Bozovic, Darko Davitkov, Milos Djuric, Vanja Krstic, Dajana Davitkov

**Affiliations:** 1 Department of Forensic Veterinary Medicine, Faculty of Veterinary Medicine, University of Belgrade, Bulevar oslobodjenja 18, 11000 Belgrade, Serbia; alexjellena@vet.bg.ac.rs (J.A.R.); nesic@vet.bg.ac.rs (V.N.); dajana@vet.bg.ac.rs (D.D.); 2 Department of Equine, Small Animal, Poultry and Wild Animal Diseases, Faculty of Veterinary Medicine, University of Belgrade, Bulevar oslobodjenja 18, 11000 Belgrade, Serbia; bozovicia@vet.bg.ac.rs (A.I.B.); milos.djuric@vet.bg.ac.rs (M.D.); vanjak@vet.bg.ac.rs (V.K.)

**Keywords:** abuse, animal, injuries, necropsy

## Abstract

**Simple Summary:**

Animal cruelty poses intricate societal challenges, carrying adverse repercussions for both the victims and broader communities. According to the Animal Welfare Law, it encompasses any deliberate actions that inflict pain, suffering, distress, or death upon an animal, marking it as socially reprehensible behavior. This study was conducted from 2014 to 2023. In this study, a total of 338 dogs and 54 cats were submitted for necropsy. Animals were received in the Department of Forensic Veterinary Medicine, Faculty of Veterinary Medicine, University of Belgrade. Diagnostic procedures—radiological, histopathological and toxicology examinations—were performed in cases where they were needed. The cause of death was defined as a primary lesion leading to death. The manner of death was categorized as non-accidental, natural, accidental (motor vehicle accident, animal bites, anesthesia-related deaths, and veterinary malpractice), or undetermined. This study showed that most of the animals that were abused were medium-sized, mix-breed dogs which were found on public property. It is important to highlight that the number of abused animals is probably much higher, but due to an inadequately developed system in animal cruelty cases, there are a large number of animals on which necropsy is not performed.

**Abstract:**

The aim of the study was to point out the importance of recognizing non-accidental injuries (NAI) and to highlight the importance of reporting such cases, as well as prosecuting the perpetrators, in order to detect potentially existing violence or prevent future violence in society. A total of 338 dogs and 54 cats were submitted for necropsy. Out of 338 dogs examined, 175 (51.8%) died due to non-natural cause of death, 122 from natural causes (36.1%), and 35 (10.3%) due to accidental injuries, and in 6 cases (1.8%) the cause and manner of death were undetermined due to advanced post-mortal changes. Out of 54 examined cats, 21 died due to non-natural causes (38.9%) and the same number of cats died due to natural causes. The prevalence of accidental injuries resulting in death were 20.4% (n = 11) and in 1.8% (n = 1) the manner of death remained indeterminate. The high number of animals confirmed to have died from unnatural causes in this study highlights the need for greater involvement from the police, prosecution, and society as a whole to reduce the number of violent animal deaths in the future.

## 1. Introduction

Cruelty towards animals is a complex and important social problem with negative consequences for the victims and as well as for society [1]. Based on Animal Welfare Law (“Official Gazette of RS” No 41/09), cruelty is defined as socially unacceptable behavior that intentionally causes pain, suffering or distress, and/or death to an animal. Acts of cruelty towards animals inflict significant harm on them. Therefore, animals should be granted protections akin to those afforded to human crime victims. Apart from the suffering caused by such cruelty, animals also face unfair treatment within the criminal justice system. For instance, animals subjected to abuse lack representation of their interests in court, with only the state having the authority to prosecute crimes against them [2]. Based on the Republic Statistical Office during the 5-year period from 2018 to 2022, there were a total of 1017 reports related to the criminal offense of killing and abusing animals. Charges were filed in a total of 121 cases, and a total of 103 individuals were convicted. In Serbia, the number of companion animals have increased in recent years, so on average, about 100,000 new puppies are registered annually in our country, and 30,000 to 35,000 are deregistered due to death or export. In 2022, a total of 93,499 dogs were microchipped, and 32,736 were deregistered. As the authors mentioned earlier, this number is not totally correct because veterinarians do not remove dead animals from database regularly and owners often do not inform veterinarians but bury them on their own. The higher number of animals is only one of the many reasons why they need more public and legal attention.

Companion animal abuse is a significant concern in our society, particularly for veterinarians and law enforcement agencies dedicated to its prevention [3]. Veterinarians have a moral obligation to report any suspected cases of companion animal abuse to the appropriate authorities [4]. Animals that have died under suspicious circumstances should undergo postmortem examination at reference laboratories staffed with experienced veterinary forensic pathologists who document necessary evidence for the further prosecution of perpetrators. A veterinary forensic pathologist also collaborates with governmental agencies responsible for investigating crimes against animals [5]. With a significant rise in the number of forensic cases submitted to diagnostic laboratories worldwide [6,7], there is a growing need for enhanced expertise in identifying non-accidental injuries, which could pose a challenge for veterinary forensic pathologists, many of whom serve as expert witnesses in court [3,8]. 

Killing and harming animals is defined as a criminal offence, based on Article 269 of the Criminal Code of the Republic of Serbia (Official Gazette of RS, No. 85/2005, 88/2005—amended, 107/2005—amended, 72/2009, 111/2009, 121/2012, 104/2013, 108/2014, 94/2016, and 35/2019). Also, in all cases of malicious poisoning due to the placement of poison in public areas (parks, streets, squares), there is a danger to children (and all people), thus constituting the criminal offense of endangering public safety (Article 278, Criminal Offenses against Public Safety of People and Property).

Despite numerous legislative acts, the killing and mistreatment of animals remain prevalent. However, precise information on the number and types of animal abuse, as well as the most common non-accidental injuries (NAI), is unknown. Due to this fact, the aim of this study is to categorize the most common types of NAI and their prevalence in companion animals during a 10-year period at the Department of Forensic Veterinary Medicine, Faculty of Veterinary Medicine, University of Belgrade, Serbia.

## 2. Materials and Methods

### 2.1. Sample Population

The study was conducted over a ten-year period, from 2014 to 2023. It encompassed dogs and cats subjected to necropsy at the Department of Forensic Veterinary Medicine, Faculty of Veterinary Medicine, University of Belgrade. A total of 338 dogs and 54 cats underwent forensic necropsy, conducted at the request of owners, public prosecutors, and/or animal protection societies.

Radiological and histopathology examinations were performed when they seemed necessary. In cases of suspected poisoning, stomach contents or liver tissues were sampled for toxicological analysis in accredited laboratories. Data pertaining to breed, sex, age, weight, microchipping, ownership, admission date, and location of discovery were recorded. Whenever feasible, information regarding the health status before the postmortem examination, including diagnostic and therapeutic procedures, was collected.

### 2.2. Cause and Manner of Companion Animal Death

The cause and manner of death were determined based on the history data, clinical symptoms, physical evidence (injuries or traces), pathological and radiological findings, laboratory analyses, and witness statements. The cause of death is defined as the primary lesion leading to death, while the manner of death is categorized as non-accidental, natural, accidental (motor vehicle accident, animal bites, veterinary malpractice, and anesthesia-related deaths), or undetermined [9,10]. Non-natural causes of death were classified as poisoning, gunshot injuries, blunt and sharp force trauma, neglect, and asphyxia. Natural causes of death were divided into infectious diseases, heart diseases, gastrointestinal disorders, and neoplastic transformations. Accidental injuries were divided into motor vehicle injuries, bite injuries, anesthesia-related deaths, and veterinary malpractice. Undetermined deaths of companion animals refer to situations where animals were in an advanced decomposition stage.

### 2.3. Statistical Analysis

After all data had been submitted, statistical analysis was performed using IBM SPSS Statistics (version 20).

## 3. Results

### 3.1. Animals

During the 10-year period (from January 2014 to December 2023), a total of 392 animals underwent necropsy, comprising 338 dogs (190 male and 148 female) and 54 cats (22 male, 32 female) (Table 1). A total of 53.5% (n = 181) of the dogs were marked with microchips, while only 1.8% of cats had an identification number (n = 1). The microchip data about sex, breed, and age were collected or described by the veterinary forensic pathologist or obtained from the owner. The most common dog breeds were mixed breed dogs, followed by German Shepherds, Staffordshire Terriers, Labradors, Pomeranians, and Rottweilers. The mean age of dogs was 3.9 years (median 3 years), with the youngest being 3 months and the oldest being 15 years. Regarding body weight, the average weight was 19.2 kg (with the smallest being 0.3 kg and the largest being 72 kg). The majority of examined cats were female (n = 32, 59.2%), mixed-breed (n = 50, 92.6%), and with the mean age of 2 years (median age 2.9 years) and average body weight 3.21 kg (with the smallest being 600 g and the largest being 8 kg). Dogs were primarily submitted by owners (61.4%), followed by public prosecutors (26.4%) and, in 6.5% of cases, by animal protection societies. A similar pattern was observed for cats, with owners submitting 61.4% of cases, public prosecutors 18.2%, and animal protection societies 3.6%.

### 3.2. Causes and Manner of Death in Dogs

**Figure 1:**
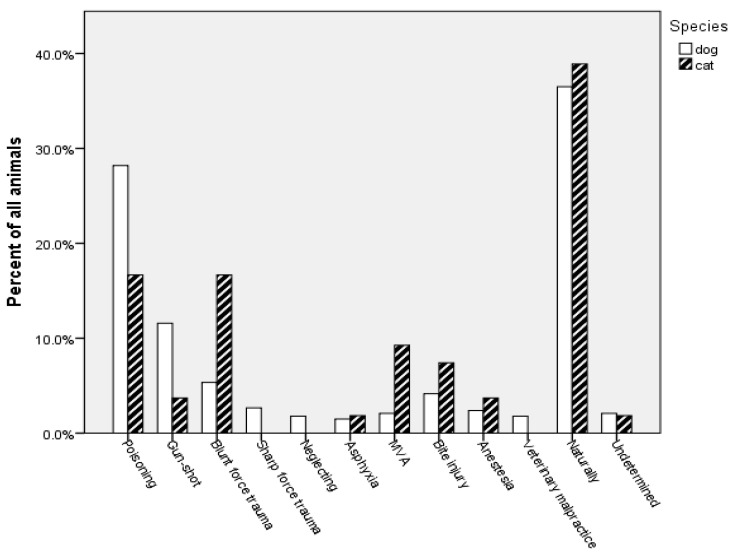
The causes of death in dogs and cats.

From the total of 338 dogs examined, 175 died due to non-natural cause (51.8%), 122 died of natural causes of death (36.1%), 35 from accidental injuries (10.3%), and in 6 cases (1.8%), the cause and manner of death could not be determinate due to postmortem changes (Figure 1).

The most common cause of non-natural death was poisoning (n = 96, 28.4%), followed by gunshot injuries (n = 41, 12.1%), blunt (n = 18, 5.3%) and sharp force trauma (n = 9, 2.7%), neglect (n = 6, 1.8%), and asphyxia (n = 5, 1.5%). From natural causes of death, the most frequent were cardiovascular diseases (n = 44, 12.9%), infectious diseases (n = 41, 12.2%), gastrointestinal disorders (n = 19, 5.6%), and neoplastic malformations (n = 18, 5.3%). The prevalence of accidental injuries was 10.4% (n = 35): motor vehicle accidents 2.1% (n = 7), 4.2% bite injuries (n = 14), 2.4% anesthesia-related death (n = 8), and 1.8% veterinary malpractice (n = 6).

**Figure 2:**
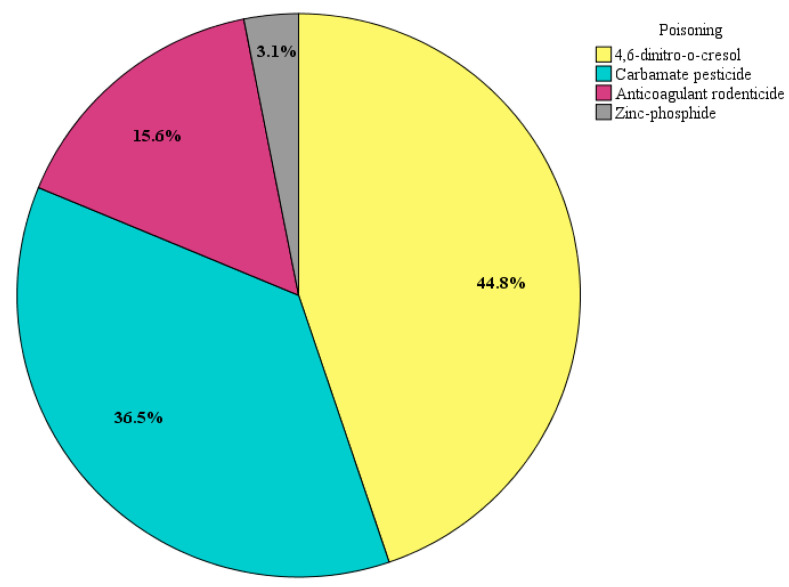
Frequency of different toxins used in dog poisoning during the 10-year period.

**Figure 3:**
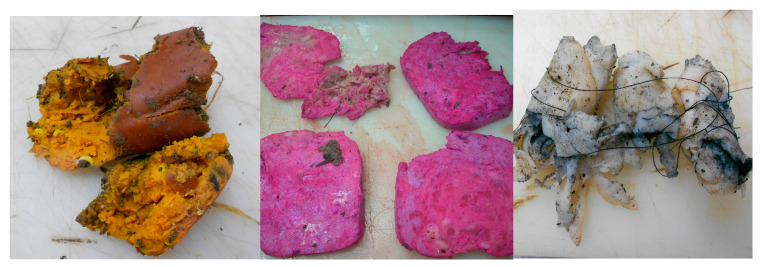
Different types of baits (1. 4,6-dinitro-o-cresol (DNOC); 2. Bromadiolone; 3. zinc phosphide).

This retrospective study ranks poisoning of dogs as the primary cause of death due to non-accidental injuries. The perpetrators most commonly used 4,6-dinitro-o-cresol (DNOC) (44.8%, n = 43), followed by carbamate pesticide (36.5%, n = 35), anticoagulant rodenticide (15.6%, n = 15), and zinc-phosphide (3.1%, n = 3) (Figure 2). The suspicion of poisoning was raised during the collection of anamnestic data, and in cases of DNOC poisoning, when there was pronounced yellow discoloration of the coat in the head region and front extremities. In most cases, suspicious substances were found in the stomach or as part of the vomit or bait that owners or the police found at the scene (Figure 3). In all poisoning cases, macroscopic changes included pronounced hyperemia of the internal organs, as well as hemorrhages, which were most pronounced in cases of anticoagulant rodenticide poisoning.

**Figure 4:**
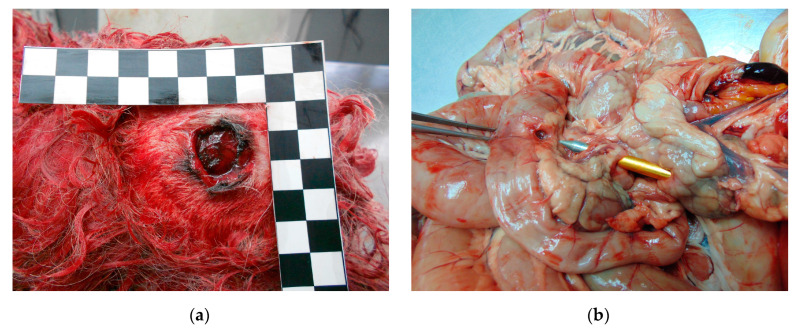
(**a**) Gunshot wound in the head region, with characteristic gunpowder residue; (**b**) pancreas and small intestine perforation with crossbow.

The second most common non-accidental injury and cause of death in dogs was gunshot injuries (n = 41, 12.1%). The most commonly used weapons were guns (46.3%, n = 19), rifles (31.7%, n = 13), air guns (19.5%, n = 8), and crossbows (1 case), respectively. The most affected body regions were the head, fore-limbs and thorax (Figure 4a), and in a case with a crossbow, the massive destruction of internal organs (Figure 4b). Massive hemorrhages due to organ rupture, blood vessel injuries and rib fractures were present in most cases. In all cases except one (infection), the mechanism of dead was hypovolemic shock due to massive bleeding.

Injuries caused by blunt force trauma were present in 18 cases (5.3%), and the main findings were subcutaneous hemorrhages, followed by spleen and liver rupture. In one case, rupture of urinary bladder was present. Sharp force trauma was present in nine cases (2.7%). Depending on the weapon used (knives, axe, hammer, etc.), different types of gross lesions were present. In seven cases of sharp force trauma, injuries were in the head region, and in two other cases, they were in the thoracic region. In one case, the owner stabbed his dog with a knife 17 times in thorax, and massive hemorrhages and injuries to the lung and heart were presented. Neglect of dogs was present in six cases (1.8%). In all cases, emaciation was present with muscular and fat tissue atrophy. Bone structures were clearly visible and decubitus was present in all joint regions. In the stomach of the two dogs, several rags and a towel were found. Death due to asphyxia was present in four cases (1.2%), there were two cases of ligature strangulation, one case of drowning, and one case of choking on a foreign object. From accidental manner of death, in the majority of bite injury cases, the bitten dogs weighed less than 15 kg, except for one dog that weighed 24 kg. These types of wounds (*vulnus punctum*, contusio) were predominantly located in the head and neck region, as well as the front extremities, and rarely in the abdominal and hind extremity regions. In one case, where a Labrador bit a medium schnauzer, typical degloving was noticed on both front limbs with necrosis. In total, seven cases (2.1%) of motor vehicle accidents (MVA) occurred. One animal was hit by a bus, two animals that were hit by a van were large animals (more than 35 kg—Labrador and German Shephard), and the other four animals hit by a car were smaller dogs (less than 15 kg). Lesions such as subcutaneous hemorrhages, hematomas, diaphragmatic rupture and, in one case, liver rupture, were present. Veterinary malpractice was present in six cases (1.8%), and in most cases in female dogs during sterilization procedures or C-sections, manifesting as bleeding due to inadequate ligature placement, infections that developed several days after surgical intervention, or ligating ureters and inducing hydronephrosis. Anesthesia-related deaths were present in eight cases (2.4%) and an undetermined manner of death was present in six dogs (1.8%).

### 3.3. Causes and Manner of Death in Cats

Out of 54 examined cats, 21 died due to non-natural causes (38.9%) and the same number of cats died due to natural causes. The majority of the non-natural deaths were poisoning and the blunt force trauma (n = 9, 16.7%), followed by gunshot injuries (n = 2, 3.7%) and asphyxia (n = 1, 1.8%). In poisoning cases, carbamate pesticides were most commonly used (six cases), then anticoagulant rodenticide (two cases) and one poisoning with DNOC. 

Similar injuries to those present in dogs that died due to mechanical force were also present in cats, although in cats they were more pronounced. In addition to subcutaneous bleeding and bleeding in the muscles, there were also rib fractures and rupturing of the parenchymal organs. Gunshot injuries were present in two cats (3.7%) and perpetrators used guns and air guns. Projectile wounds were present in the head and in the chest region, respectively. Death due to asphyxia was present in one case (1.8%) and the manner in which asphyxia death injuries occurred was asphyxia due to CO intoxication. The most frequent natural causes of cats′ deaths were infectious diseases (n = 12, 22.2%), followed by cardiovascular diseases and neoplastic malformations (each n = 4, 7.4%). Gastrointestinal disorders were present in one case (1.8%). Deaths caused by motor vehicle accidents were present in five cases (9.3%). Massive hemorrhages with rib fractures, rib displacement, pneumothorax, hemoabdomen, and limb fractures were present. Bite injuries in four cats were caused by dogs (7.4%). Unlike the injuries observed in dogs, deep penetrating wounds in cats were localized throughout the body, most commonly in the thoracic region. All four animals died due to bleeding caused by perforation of the lungs or blood vessels. Anesthesia-related deaths were present in two cats. In one case, the cause and manner of death could not be determinate because of the poor body condition.

## 4. Discussion

The field of veterinary forensics is still developing in many countries [9,11]. In some countries, as well as in Serbia, the number of papers in the field of veterinary forensics and animal abuse cases is still insufficient. In instances where animal abuse is suspected, the involvement of clinicians and veterinary forensic pathologists is pivotal, requiring ongoing education and training. Additionally, many countries lack established protocols for handling different forms of non-accidental injuries, which would ensure impartiality and accountability throughout legal proceedings. These protocols would facilitate the documentation, analysis, and interpretation of evidence by a diverse range of experts, including veterinarians, law enforcement, and prosecutors, involved in addressing instances of animal cruelty. Implementing such protocols would establish standards and streamline prosecution processes, potentially leading to more stringent penalties for offenders. In addition to the missing protocols, it is crucial to conduct adequate examination and sampling at the crime scene. Considering this crucial deficiency, a lot of information about a case, which could be relevant for diagnosis, is often lost. In Serbia, as in many other countries, there is insufficient interest from the parties involved in the process, and first and foremost the police, who are often a key factor in resolving these criminal acts. 

During this study, a higher number of animal abuse cases was observed in dogs compared to cats (51.8% dogs, 38.9% cats), which is in accordance with other studies [12,13]. The reason for this is the fact that dogs are more visible (often being walked by their owners), which could lead to a higher number of cruelty reports involving dogs, and because owners are often present when an accident occurs. On the other hand, cats frequently live outside, and in most cases, after accidents they cannot be found. More male dogs were submitted for necropsy than female dogs, but no statistical significance was noted (56.2% male, 43.8% female). However, other studies have noted more females being autopsied [7,11,14]. In cats, more females (59.2%) than males (40.8%) were submitted for necropsy. This result is not in accordance with other results, where male cats were dominant [6]. The smaller number of cats compared to dogs in animal cruelty cases can be explained by the fact that when they are injured, cats tend to go to distant places and usually do not have a microchip, which makes them difficult to find. In other studies [3,6,7], although higher numbers of dogs were examined, the ratio between dogs and cats was not so high as it was in our study. Such a study is that of Rebollada-Merinuo, where 57.29% dogs and 42.71% cats were examined. Other authors noticed that almost half of the dogs did not have a microchip [7], and in our study 53.7% of the dogs had microchips, while only 1.8% of the cats were microchipped. Usually, owners do not microchip cats because they think that their animal does not have to have a microchip if it never leaves the flat. This is a big issue to work on and it is an important task to raise awareness because a pet owner is responsible, obligated by Veterinary Law (“Official Gazette of RS”, No 91/2005, 30/2010, 93/2012 and 17/2019), for microchipping all dogs and cats at the time of their first vaccination. Similar results were noticed in other studies and present a problem in many countries, especially where a lot of stray dogs and cats are present [11,15]. The most commonly abused companion animals were mixed-breed dogs, which is expected considering the fact that they are most common breed in the country and that they are often stray dogs, which is in accordance with other studies [7,11,16]. 

In most of the cases, age was determinate by tooth examination or by reading the data from a microchip. The mean age of dogs was 3.9 years (median 3 years), with the youngest being 3 months and the oldest being 15 years old. Like in the other studies, the average age was 3.9 years, which is similar to the study by Rebollada-Merino et al. (2020), where the average age was 3 years and 1 months. When it comes to cats, the average age for cats (2.9 years) was much lower than in dogs. This is not in accordance with studies of Rebollada-Merino et al. (2020), where the average age for cats was higher, but in studies by Almeida (2018), the average age was lower than it was in this study. Regarding body weight, the average weight of dogs was 19.2 kg (with the smallest being 0.3 kg and the largest being 72 kg). This is higher than expected, because in most cases smaller animals are more often abused. Small and mid-sized animals are easier to manhandle, so probably that is the reason why they are usually victims of abuse [12].

Intoxication was the main cause of death in dogs, encompassing 28.4% of examined dogs. Poisoning is a serious problem, not just in Serbia, but also in other European countries such as Greece, Italy, Spain, France, and Belgium [17]. The reason for this high number of poisoning cases is that the number of stray dogs in Serbia is rising every year and is now estimated to be around 400,000. This number reflects the significant challenge the country faces in managing its stray dog population, which includes issues related to inhumane treatment and inadequate resources for animal welfare. Dogs are more vulnerable to poisons than cats, which has been proven in other studies [18,19,20,21]. The main reason for higher poisoning prevalence in dogs is that they are more voracious, curious about food, and love to try everything [22,23], and cats are more cautious; they do not eat everything and do not like to play with potential food. In our study, the prevalence for cats was mostly according to other papers (16.7%), but in some countries such as Brazil and the USA, the prevalence was higher [24,25]. 

Our retrospective studies pointed out that in most of the cases of malicious animal poisoning, perpetrators used pesticides banned in the Republic of Serbia—DNOC (2003), carbofuran (2014), and methomyl (2020). Despite this, the perpetrators somehow managed to acquire them and use them for malicious purposes. Although poisoning emerged as the most common cause of death in dogs in this study, it is important to note that the number of reported suspicions of poisoning is much higher. In order to facilitate more effective prosecutions of perpetrators, who are often difficult to link to the abuse of toxic substances, it is necessary to implement stricter control measures during the sale of agricultural equipment. Additionally, to monitor the prevalence of this issue in our society, it is essential to establish a central registry of confirmed poisoning cases of both owned and stray dogs. This registry would allow the registration of offenders involved in this type of cruelty toward animals [26,27]. 

**Figure 5:**
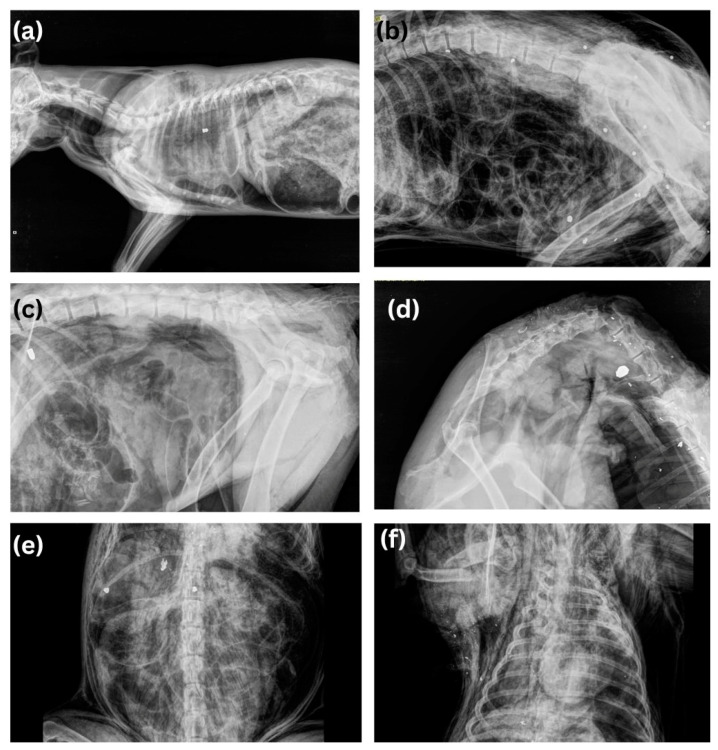
Radiographs of different weapons used in illegal animal shooting: (**a**) ai5 gun, (**b**) shotgun, (**c**) gun, (**d**) rifle, (**e**) gun ammunition destroyed by bone, (**f**) “snowstorm” in gunshot wound where projectile exits the body.

Gunshot injuries were the second most common cause of the death in dogs and in cats, after poisoning and blunt force trauma. In rural areas, rifles or shotguns were most commonly used, but in urban areas it was guns that were more common. Calibers such as 0.22 LR (Long Rifle)-caliber weapons are also present and popular in Serbia. This caliber is known for its accuracy, low recoil, and low cost of ammunition, making it ideal for recreational shooting, shooter training, and pest control. These 0.22 LR-caliber weapons include various types of rifles and pistols and are widely available in Serbia; it is also a global caliber [28]. In most cases, the affected regions were the head (usually gun), forelimbs and thorax (in shotgun cases). When an air gun was used, injuries were present mostly in the thorax or abdomen. Air rifles are very common in Serbia, and are increasingly used due to their easy accessibility, price, and the fact that rifles with speeds over 200 m/s and with lead bullets can be purchased, which have great penetrating power. In all cases, death by air-rifle was indicated by the retention of the characteristic projectile in the carcass at the time of postmortem and identified by radiology [29]. Radiology is an indispensable method in all cases where there is suspicion of injury, both for diagnostic purposes and for identifying injuries to bone structures and soft tissues. (Figure 5) In this study, like in the study by Araujo et al., (2021) most of the animals were shot on public property, and only two animals were shot during hunting [30].

In most papers, blunt force trauma was the prevalent non-accidental injury [7,12], and in our study it came in third place in dog cases (18 dogs) and in first place, together with poisoning, in cats (9 cats). The explanation for the lower number of cases of this type of mechanical injury compared to other studies [7,12] could be that motor vehicle accidents (MVAs) were analyzed separately and classified as accidental injuries. Most commonly, blunt force trauma occurred due to physical aggression to animals, usually in the region of the head or thorax. This can be indicative of perpetrators’ malicious intent, and not just the intention to scare the animal, because injuries in the head region are more likely to be fatal. It is also important to emphasize that a large number of animals that die due to blunt mechanical force or as a result of being hit by a motor vehicle often do not undergo autopsy. Instead, their owners bury such dogs or, in the case of strays, they are taken away by the municipal hygiene service for incineration.

In addition to blunt force trauma, sharp force trauma was also noticed, but statistically in a smaller number (nine dogs). The most commonly used weapons were a knife, axe, or hammer. Interestingly, in most of the cases dogs were mid-sized, and there was just one case of a smaller dog. To apply sharp force trauma, a perpetrator needs to be close to the animal, so this excludes the possibility of animals’ aggressive and perpetrators’ self-defense behavior.

When it comes to law enforcement, based on the Republic Statistical Office during the 5-year period from 2018 to 2022, in cases of killing animals and animal abuse, charges were filed in a total of 121 cases, and a total of 103 individuals were convicted (total of 1.017 reports in five years). These numbers are extremely low, considering that in the United Kingdom, animal welfare organizations such as the RSPCA (Royal Society for the Prevention of Cruelty to Animals) and other organizations collect data on reports of animal cruelty and, according to RSPCA data, over 100,000 cases of animal cruelty are reported annually in England and Wales. This points to the fact that a small number of cases of killing and abuse are reported annually, highlighting the need to raise awareness about the prevalent violence against animals.

In order to identify risk factors in all cases of NAI for animals it would be of great interest to collect data from owners about social, economic, and psychological aspects. Based on this information, it would be possible to adequately recognize cruelty and provide forecasts.

## 5. Conclusions

Companion animals are in the first place living beings able to feel pain, fear, and suffering, and they have a significant role as companions in human life. Based on the results of this and other, similar studies, cruelty toward animals is a global problem, the prevention of which is based on educating and raising awareness among citizens about the rights and needs of animals; improving and enforcing laws regarding their welfare; promoting responsible ownership (microchipping, sterilization, neutering, immune-prophylaxis); and developing strategies to prevent animal abuse and killing, as well as by reporting such cases to the relevant authorities.

The lack of education among veterinarians, poor crime scene processing, prosecutorial disinterest, and challenges in guiding owners toward evidence collection are all significant issues that represent just some of the problems when it comes to animal cruelty cases. The authors believe that the number of violent deaths is much higher, unfortunately; due to the sheer volume of data, many cases are not prosecuted to completion.

## Figures and Tables

**Table 1:** Total number of dogs and cats which were examined in the ten-year period.

			Year										Total
			2014	2015	2016	2017	2018	2019	2020	2021	2022	2023	
Species	**Dog**	**Count**	**10**	**20**	**25**	**26**	**31**	**28**	**56**	**45**	**30**	**67**	**338**
		% dog	3.0%	5.9%	7.4%	7.7%	9.2%	8.3%	16.6%	13.3%	8.9%	19.8%	100.0%
		% of Total	2.6%	5.1%	6.4%	6.6%	7.9%	7.1%	14.3%	11.5%	7.7%	17.1%	86.2%
	**Cat**	**Count**	**5**	**3**	**2**	**5**	**4**	**6**	**9**	**9**	**3**	**8**	**54**
		% cat	9.3%	5.6%	3.7%	9.3%	7.4%	11.1%	16.7%	16.7%	5.6%	14.8%	100.0%
		% of Total	1.3%	0.8%	0.5%	1.3%	1.0%	1.5%	2.3%	2.3%	0.8%	2.0%	13.8%
**Total**		**Count**	**15**	**23**	**27**	**31**	**35**	**34**	**65**	**54**	**33**	**75**	**392**
		% of Total	3.8%	5.9%	6.9%	7.9%	8.9%	8.7%	16.6%	13.8%	8.4%	19.1%	100.0%

## Data Availability

The data presented in this study are available on request from the corresponding author.
